# Cellular feedback dynamics and multilevel regulation driven by the hippo pathway

**DOI:** 10.1042/BST20200253

**Published:** 2021-08-10

**Authors:** Jiwon Park, Carsten Gram Hansen

**Affiliations:** 1 University of Edinburgh Centre for Inflammation Research, Institute for Regeneration and Repair, Queen’s Medical Research Institute, Edinburgh bioQuarter, 47 Little France Crescent, Edinburgh EH16 4TJ, U.K.; 2 Institute for Regeneration and Repair, University of Edinburgh, Edinburgh bioQuarter, 5 Little France Drive, Edinburgh EH16 4UU, U.K.

**Keywords:** cell migration, feedback, hippo pathway, mitosis, STRIPAK, YAP/TAZ

## Abstract

The Hippo pathway is a dynamic cellular signalling nexus that regulates differentiation and controls cell proliferation and death. If the Hippo pathway is not precisely regulated, the functionality of the upstream kinase module is impaired, which increases nuclear localisation and activity of the central effectors, the transcriptional co-regulators YAP and TAZ. Pathological YAP and TAZ hyperactivity consequently cause cancer, fibrosis and developmental defects. The Hippo pathway controls an array of fundamental cellular processes, including adhesion, migration, mitosis, polarity and secretion of a range of biologically active components. Recent studies highlight that spatio-temporal regulation of Hippo pathway components are central to precisely controlling its context-dependent dynamic activity. Several levels of feedback are integrated into the Hippo pathway, which is further synergized with interactors outside of the pathway that directly regulate specific Hippo pathway components. Likewise, Hippo core kinases also ‘moonlight’ by phosphorylating multiple substrates beyond the Hippo pathway and thereby integrates further flexibility and robustness in the cellular decision-making process. This topic is still in its infancy but promises to reveal new fundamental insights into the cellular regulation of this therapeutically important pathway. We here highlight recent advances emphasising feedback dynamics and multilevel regulation of the Hippo pathway with a focus on mitosis and cell migration, as well as discuss potential productive future research avenues that might reveal novel insights into the overall dynamics of the pathway.

## Introduction

**Figure 1.**
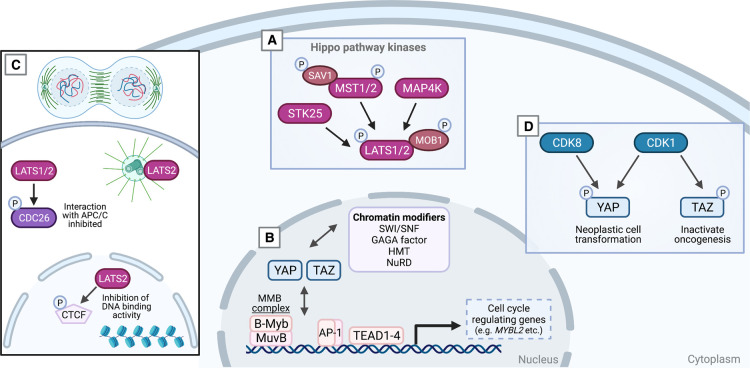
Regulation of mitosis by the Hippo pathway and YAP/TAZ. (**A**) Upstream Hippo pathway serine/threonine kinases control activation of LATS1/2 kinases, which in turn directly phosphorylate YAP/TAZ on multiple serine residues and thereby controls YAP/TAZ transcriptional activity though regulating their subcellular localisation. (**B**) In the nucleus, YAP/TAZ form transcription complexes with various transcription factors to induce expression of cell cycle regulating genes. The cell cycle is further modulated by YAP/TAZ through its interaction with chromatin-modifying proteins, such as nucleosome remodelling and deacetylase (NuRD) complex and histone methyltransferase (HMT) complex, which promote remodelling of chromatin configuration to control gene transcription programmes. (**C**) LATS1/2 kinases modulate cell cycle exit during late anaphase or telophase through phosphorylation of CDC26 and CTCF. (**D**) Cyclin-dependent kinases provide an additional level of regulation of mitosis through direct phosphorylation of YAP and TAZ.

The Hippo pathway was initially identified through genetic loss of function mosaic screens in the fruit fly, *Drosophila melanogaster* [1–6]. The evolutionary conservation from flies to humans is extensive, although key differences, including gene duplications, exist [7]. The signalling pathway consists of a regulatory serine/threonine kinase cascade that when activated ultimately phosphorylate the co-transcriptional regulators YAP and TAZ [7]. YAP was originally identified independently as an interactor of Yes kinase [8], acidic oligomeric 14-3-3 proteins [9] and as a cofactor of the TEAD transcription factors [10]. TAZ was also isolated as a 14-3-3 protein family-binding protein [11]. Since these original findings, a substantial interest in YAP and TAZ and the biology that they drive has evolved. Seminal studies identified MST1/2, STK25 and the MAP4K family of kinases as activators of the large tumour suppressor kinase1/2 (LATS1/2) [12–16] (Figure 1A). LATS1/2 belong to NDR family of serine/threonine kinases and directly phosphorylate YAP and TAZ on multiple sites. This consequently cause YAP and TAZ to predominantly localise to the cytoplasm and thereby inhibits YAP/TAZ. As YAP and TAZ do not contain DNA-binding domains, their transcriptional activity is dependent on interaction with cognate nuclear transcription factors, predominantly the transcriptional enhanced associate domain (TEAD) family [10,19–21]. Consequently, the dephosphorylation (and thereby inactivation) of upstream components of the Hippo pathway kinase cascade is critical for YAP/TAZ transcriptional activity. The supramolecular PP2A-STRIPAK (Striatin-interacting phosphatase and kinase) complex is central for this regulation [22–25]. This noncanonical phosphatase complex contains both kinase and phosphatase subunits for enhanced regulation, which allows dynamic cellular regulation upon diverse cellular stimuli and functions as an adaptable signalling centre [22]. STRIPAK complexes PP2A to multiple Hippo components, such as the scaffolding protein NF2 as well as LATS1/2 activating kinases [14,24,26–29]. PP2A-STRIPAK consequently activates YAP and TAZ through dephosphorylation and suppression of the upstream Hippo pathway kinase module [14,24,26–30]. The Hippo pathway is a central cellular signalling nexus and regulated by polarity, cell density, machanotransduction and diffusible chemicals [7,31]. STRIPAK-YAP/TAZ signalling is controlled by a range of stimuli, including activation of GPCR signalling by ligands, such as the exemplar Hippo pathway regulator lysophosphatidic acid (LPA) [18,30]. The bioactive lipid LPA activates the STRIPAK complex, which turns off the Hippo pathway kinase cascade [18,30,32,33]. Deactivating STRIPAK inactivates YAP/TAZ activity and reduces tumorigenic potential and elevated expression of STRN3 and STRN4, which function as recruiting STRIPAK factors for LATS activating kinases, are a prominent feature in some cancers [25,26,34–36]. However, PP2A activity is widely decreased in a range of cancers and PP2A activators show therapeutic promise [37,38]. Importantly, additional levels of nuclear YAP/TAZ regulation exist. This additional regulation takes place both via nuclear Hippo pathway independent phosphorylation of YAP/TAZ, such as upon energy stress via AMPK mediated phosphorylation of YAP [39,40], and apparent phosphorylation independent mechanisms [41–43]. The critical role of Hippo pathway signalling in decision-making processes in almost all types of fundamental cell biology has spurred a great interest into this pathway. A precise, dynamic and robust regulation of the pathway is necessary, as otherwise developmental defects [44,45], fibrosis [46,47], impaired regeneration and cancer [30,48–51] occur. This precise regulation is obtained through multiple spatio-temporal level feedback, including transcriptional induction of upstream negative regulators, such as *LATS2*, *AMOTL2, CAV1* and *NF2* [17,20,52–59]. This multilevel response consequently reinforces and feeds robustness into the overall regulation of the pathway. Here, we highlight instances of fundamental cellular processes, using mitosis and cell migration as exemplars, that involve multilevel Hippo pathway-mediated integration. This dynamic complexity and synergistic feedback likely function to tightly regulate and safeguard central cellular processes.

## Mitosis

Control of cell number is essential to animal development, regenerative processes and in tissue homeostasis. Consequently, dysregulation of cell number may result in tumour formation, developmental defects or organ failure. The Hippo pathway regulates cell number by modulating cell proliferation, cell death and cell differentiation. These functions are shared and evolutionarily conserved from *Drosophila* to mammals [60]. Chromatin topological organisation is instrumental in gene transcription and overall chromosome compartmentalisation has emerged as critical to higher-order genome organisation [61,62]. During cell division, nuclear chromatin undergoes marked changes with respect to shape and degree of compaction [63–65]. Once segregated by the spindle, chromatin decondenses to re-establish its interphase structure competent for DNA replication and transcription. The precise mitotic chromatin condensation and decondensation is therefore a highly regulated process for mitotically active cells [63]. The Hippo pathway and YAP and TAZ contribute to the regulation of mitosis through interaction with transcription factors, chromatin modifiers and by inducing expression of mitotic genes.

YAP and TAZ modulate mitosis through the formation of a transcriptional complex with TEAD and AP-1, which promote proliferation and regulate expression of a range of cell cycle genes, including genes driving G1/S phase transition, DNA replication and quality control [20,66–70]. Moreover, YAP induces the expression of the transcription factor MYM proto-oncogene like 2 (B-MYB) (encoded by *MYBL2*) [71], a subunit of the multi-protein Myb-MuvB (MMB) complex. The MuvB core (composed of LIN9, LIN37, LIN52, LIN54 and RBBP4) upon entry into the cell cycle dissociates from p130 in the mitotic repressive DREAM complex [72] and binds to B-Myb during S phase to activate transcription of genes expressed late in the cell cycle. Consequently, overexpression of B-Myb shifts this equilibrium in favour of the mitosis promoting MMB complex and disrupts the DREAM complex [72,73]. Hyperactivation of B-Myb, therefore, cause elevated rate of mitosis and hyperproliferation in a range of cancers [74]. In addition to transcriptionally inducing *MYBL2* (encoding B-Myb), YAP/TAZ also complex with B-Myb, which facilitate Myb-MuvB (MMB) chromatin binding and activation of the complex [71,75–77] (Figure 1B). Additional Hippo pathway-mediated regulation of the MMB complex takes place via LATS2. LATS2 phosphorylates and thereby activates the dual-specificity serine/threonine and tyrosine kinase DYRK1A, which in turn phosphorylates the LIN52 subunit of MuvB and consequently promotes the assembly of the DREAM complex [78,79]. Of note, global phosphoproteomic studies in glioblastoma cells highlight that DYRK1A may regulate the Hippo pathway, including via phosphorylation of NF2 and MAP4K4 [80]. Similarly, in flies the DYRK-family kinase Minibrain (Mnb) promotes Yorkie (Yki), the fly ortholog of YAP and TAZ-dependent tissue growth [81]. In addition to mediating their activity via transcription factors, YAP/TAZ also interact and function with multiple chromatin-modifying proteins, including SWI/SNF, GAGA factor, Mediator complex, Histone methyltransferase complex and the NuRD complex [82]. Consequently, depending on the cellular context, YAP/TAZ dynamically interact with a wide array of transcriptional regulators to control cell proliferation. However, the exact mechanism of how this process is fine-tuned remains to be elucidated.

The Hippo pathway additionally regulates mitosis via the zinc finger transcription factor CCCTC-binding factor (CTCF). CTCF is partially retained on mitotic chromosomes and immediately resumes full binding in ana/telophase [83–86]. CTCF and CTCF motifs function as *cis*-elements and therefore provide critical roles in nucleosome positioning. Consequently, this governs the inheritance of nucleosome positioning at regulatory regions throughout the cell cycle, especially those associated with fast gene reactivation following replication and mitosis [84]. LATS2 likely translocate between the nucleus and the cytoplasm, and a visible fraction localises to the centrosome [87–89]. LATS kinases phosphorylate CTCF in the zinc finger (ZF) linkers and disable its DNA-binding activity [90] (Figure 1C). Chromatin structural transitions during mitosis are tightly controlled as perturbances in this dynamic process can lead to genome dysfunction and culminate in loss of cellular fitness [63,91]. While YAP/TAZ interact with transcription factors to regulate mitotic entry, LATS1/2 exert negative feedback at the cytoplasmic level through direct inhibition of YAP/TAZ, as well as at the nuclear level through disassociation of CTCF from chromatin domains containing YAP target genes [90].

The core Hippo pathway kinases LATS1 and LATS2 further bind to and regulate additional master regulators of mitotic exit. LATS1 binds CDC25B [92] and LATS1/2 phosphorylate CDC26 (known as APC12) [93] (Figure 1C). Phosphorylation of CDC26 inhibits the interaction between CDC26 and anaphase-promoting complex 6 (APC6) and thereby modify the tetratricopeptide repeat subcomplex APC/C, which promotes mitotic exit through reduction in cyclin-dependent kinase (CDK) activity [93,94]. CDKs additionally regulate mitosis via YAP [95] and TAZ [96] as YAP and TAZ are directly phosphorylated on multiple residues by CDK1. CDK1-mediated TAZ phosphorylation inactivates TAZ oncogenic activity [96], whereas CDK1-mediated YAP phosphorylation induces neoplastic cell transformation [95] (Figure 1D). Interestingly, it appears that this mitotic phosphorylation of YAP and TAZ cause functionally opposite cellular responses, and this, therefore, appears to be an example where YAP and TAZ divergence exist. Additionally, CDK8 directly phosphorylate YAP and promote its activation and support tumour growth in colon cancer cells [97] (Figure 1D). The precise details and intricate network of how CDKs mediate YAP/TAZ phosphorylation during mitosis is currently not fully understood [95–97]. Importantly, Hippo pathway activity is critical for cytokinesis, as cytokinesis failure triggers small G protein-mediated activation of the Hippo pathway tumour suppressor kinase LATS2 [98]. LATS1/2-deficient cells or overexpression of YAP/TAZ cause spindle and centrosome defects, which result in failures in correct chromosome segregation and highlights the role of LATS1/2 in coordinating accurate cytokinesis [87,95,96,99,100]. In addition, CDK1 phosphorylates and activates LATS upon microtubule disruption and subsequent mitotic stress induced by chemicals. This genotoxicity causes LATS to stabilise replication forks by controlling CDK2-mediated phosphorylation of BRCA2 [101,102]. CDK1 activity maintains adhesion during interphase [103], which might provide an additional level of CDK1-mediated YAP/TAZ regulation during mitosis [104]. Furthermore, through an apparent transcription-independent mechanism, phosphorylation of YAP by CDK1 is required for tight control of cytokinesis [105]. YAP co-localises to the central spindle and midbody ring with proteins required for cell contraction as well as the polarity scaffold protein PATJ [105]. YAP is therefore required for accurate cytokinesis and cell division to occur. It is worth noting that YAP is not essential for mitosis as multiple proliferative cell model systems have been genetically engineered to be without YAP, as well as some *in vivo* systems such as the zebrafish develop to adulthood and are fertile. In brief, the cell cycle affects YAP and TAZ through both LATS1/2, CDK1 and CDK8-mediated phosphorylation and coordination of these kinases are central to implement upstream signals for precise timely progression through the cell cycle. This dynamic multilevel regulation of YAP/TAZ and the LATS kinases likely ensures fidelity and robustness and thereby safeguard the timing of proper cell division [89,91,98,101,106–108].

## Cell migration

Directional cellular migration is essential during embryo development and is necessary for tissue homeostasis, such as epithelial turnover and in regenerative processes [109,110]. Since original studies carried out more than 110 years ago in both developing and injured embryonic chick embryos [111], the need to discover underlying molecular and mesoscale level mechanisms has been evident. Since then, discoveries obtained from live-cell imaging, *in vivo* model systems, biochemistry and ‘omics’ approaches have provided remarkable insights into both single and collective cellular migration to obtain multicellular mesoscale migration [109,110,112,113]. Migration and navigation through diverse three-dimensional environments require orchestrated and temporal change in cellular shape and directional movement of distinct cell populations [109,110,113]. Cell migration takes place both as individual cells, but also as collective migration driven by cell–cell contacts and directed by both chemical and biophysical cues [109,110,112,113]. Collective cell migration of epithelial cells requires coordination of actin cytoskeleton dynamics and YAP/TAZ activity, which is driven by YAP-mediated feedback interactions that involve down-regulation of E-cadherin and activation of Rac1 [114–116]. Interestingly, E-Cadherin is a prominent upstream cell–cell junction regulator of YAP/TAZ in mammalian epithelial cells [115,116].

**Figure 2.**
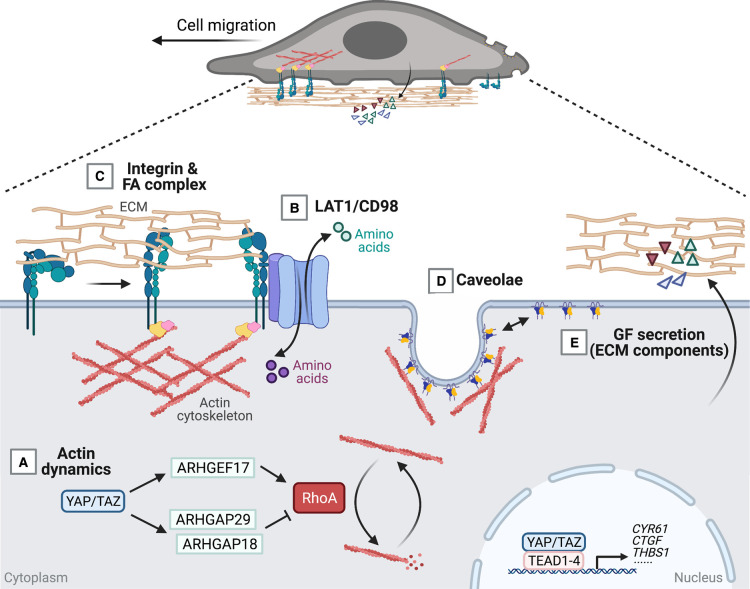
YAP/TAZ activity is central to coordinating cell migration. Cell migration requires the coordination of various plasma membrane components that link extracellular matrix to the cell cytoskeleton. (**A**) YAP/TAZ influence the dynamics of the actin cytoskeleton through the expression of Rho GTPase-activating proteins (ARHGAP18, ARHGAP29) and Rho guanine exchange factor (ARHGEF17), which in turn modulates RhoA activity. (**B**) LAT1/CD98 is a disulfide-linked heterodimer composed of SLC3A2 and SLC7A5 that promotes cell migration and survival by coupling nutrient availability and integrin activity. LAT1/CD98 exports glutamine in exchange of importing amino acids, such as leucine, isoleucine and arginine. SLC3A2 of the LAT1/CD98 heterodimer binds integrins and amplifies integrin-mediated signalling. (**C**) At the leading edge of the cell, activation of integrin receptors through binding of extracellular matrix (ECM) components promotes focal adhesion complex assembly and the contractile forces generated by the actin cytoskeleton enables movement of the cell. (**D**) Plasma membrane subdomain caveolae expression is regulated by YAP/TAZ and plays a mechanoprotective role by flattening in response to mechanical forces, such as cellular stretching and osmotic swelling. In migrating cells, caveolae frequently localise to the rear due to low membrane tension. (**E**) YAP/TAZ-TEAD-mediated transcriptional activity promotes the production of matricellular proteins, such as CYR61 and CTGF. These signalling factors promote cell adhesion, migration and proliferation.

The actin cytoskeleton is central to cell migration [117]. Dynamic polymerisation of actin monomers (G-actin) allow the generation of polar and branched actin fibres (F-actin) [117]. This process is spatiotemporally controlled by nucleation and elongation factors [117]. Consequently, actin polymerisation, retrograde actin network flow, treadmilling and contractility greatly mediated by the actin motor protein MyosinII play central roles in cell migration [110,117]. Cell migration integrates both context and cell type-specific chemical, cell–cell and cell–extracellular matrix (ECM) stimuli [109,110,113]. As a result, aberrant cell migration causes developmental defects, impaired regenerative processes and metastasis [109,110,113]. The Hippo pathway via the transcriptional mediators YAP/TAZ-TEAD function as a cellular rheostat for cellular migration, as it incorporates, mediates and dictates the necessary feedback to provide the cellular dynamics for directional migration [18,21,55,114,118–125]. Molecularly this feedback take place at several levels, which include sensing and integrating events at the plasma membrane and via the cytoskeleton [12]. YAP/TAZ are at the core of cue integration required for actin dynamics and cell migration while dysregulation of these dynamics allow cancer cells to change shape, invade surrounding tissues and metastasis [21,122–124,126]. YAP/TAZ are activated by F-actin-mediated mechanical tension and this YAP/TAZ tension sensing is inhibited by actin polymerisation inhibitor latrunculin B. Inhibition of F-actin formation causes LATS1/2 to complex with scaffolding proteins, become activated and subsequently inactivate YAP/TAZ [104,127,128]. This indicates that F-actin inhibits, and G-actin potentially facilitates this complex formation. RhoA regulates the level and dynamics of F-actin depolymerisation, such as increasing cytoskeleton rigidity and promoting a metastatic phenotype. YAP directly regulates actin dynamics through transcriptional up-regulations of Rho GTPase-activating proteins (GAPs) *ARHGAP29* and *ARHGAP18* [124,129,130], which suppresses RhoA activity and thereby destabilises F-actin. In contrast, YAP/TAZ can also induce Rho guanine exchange factor (RhoGEF) ARHGEF17 that activates RhoA activity [131] (Figure 2A). This combined escalates G- and F-actin turnover and promotes migration [124]. Thus, actin cytoskeleton dynamics regulate YAP/TAZ activity but YAP/TAZ also provide feedback into this modulation through the expression of actin regulating factors.

Plasma membrane receptors and subdomains are critical in transducing changes in the extracellular environment to cellular effects. YAP/TAZ-TEAD induce ECM receptors including the expression of the heterodimeric CD98/LAT1 [132] (encoded by *SLC3A2* and *SLC7A5*), a dual function amino-acid transporter and integrin coreceptor, which thereby link mechanotransduction directly to cellular metabolism [132–136] (Figure 2B). Moreover, focal adhesions (FAs) together with integrins, which is integral to linking the ECM to the cytoskeleton, serve as components of the FAs spatio-temporal dynamics [119,137]. YAP/TAZ-TEAD through the generation of (FA) docking proteins and regulators (de)sensitise the dynamic transmission of forces between the cytoskeleton and ECM and thereby mediates the changes in cellular tension necessary for migration (Figure 2C). In addition to plasma membrane receptors, YAP/TAZ-TEAD are also essential for the expression of the machanotransductive and endocytic competent plasma membrane domains termed caveolae [56,57]. Caveolae [138,139] locate and form in migrating cells to the rear due to low membrane tension where they activate RhoA and control contractibility [140] (Figure 2D). Cells genetically modified to have no caveolae consequently have directional cellular migration defects [140,141].

The Hippo pathway also interacts with additional cytoskeletal regulators and is modulated by multiple levels of mechanical stimuli, including shear stress responses, cell–cell interactions, and the stiffness of the microenvironment [31,48]. YAP/TAZ-TEAD regulate the expression and secretion of a range of ECM components and additional growth factors, such as AREG, CYR61, CTGF and THBS1 [20,142–144] (Figure 2). This allows the formation of chemoattractant gradient that promotes cell migration while relaying extracellular mechanical signals to transcriptional regulation (Figure 2E). In addition to secretory extracellular factors, the mechanical extracellular environment regulates Hippo pathway activity. Upon low stiffness Ras-related GTPase (RAP2) binds to and activates ARHGAP29, which results in MAP4K and subsequent LATS1/2 activation. Consequently, YAP/TAZ are inhibited and RAP2 serves as an intracellular mechanosensor to relay stress from the ECM [143]. This combined integration with the actin cytoskeleton likely fine-tune a spatiotemporally co-ordinated actomyosin system necessary for directional cell migration.

## Outlook

The Hippo pathway contains several levels of regulatory and feedback mechanisms. Prominent examples of feedback mechanisms regulating YAP/TAZ activity takes place through transcriptional up-regulation of *LATS2*, *AMOTL2* and *NF2* [55,58,59,145], modifiers of the actin cytoskeleton and plasma membrane components, such as caveolae [56,57]. It is evident that multiple levels of both positive and negative cellular feedback regulation are centred on the Hippo pathway in most, if not all, central cellular processes. Here, we have focussed on two of these processes, mitosis (Figure 1) and cellular migration (Figure 2). Many more examples are described elsewhere, such as integration of processes and organelles at the plasma membrane [12], cell size [132], mechanotransduction [31], fibrosis [46,47], cell polarity [146–148], metabolism [40,132,134–136,149], integration with auto- [150] and mito-phagy [151], in great part through interaction with numerous other cellular pathways [7]. These multilevel feedback loops are likely in place to dampen noise and prevent signal fluctuations, and to further integrate inputs from the cellular microenvironment [31,152–154]. An interesting initial perplexing observation is that many types of solid cancers are addicted to hyperactive YAP/TAZ [52,155]. However, it is well established that the pathway has an exceptionally low general somatic point mutation load [156], which means that the course to oncogenesis is currently not fully established. Corruption of the integrated and multilevel cellular feedback and regulation is therefore a likely cause as it offsets cellular and tissue homeostasis, which can result in developmental defects, fibrosis or cancer [30,44–51]. These dynamic molecular couplings serve in non-pathological conditions as flexible integrators of context-dependent stimuli, and likely provide robustness and thereby safeguard fundamental cellular processes from failing. YAP/TAZ, therefore, regulate both the expression of receptors and prominent signalling molecules, including chemical and matrix components [7,12]. YAP/TAZ play a central role by changing both the microenvironment chemical gradients and the intracellular response to already established physiochemical gradients within the microenvironment. New insights will undoubtedly be gained by implementing genetically encoded tagged versions of the Hippo pathway components expressed via genome-editing strategies to allow for the expression of these versions at endogenous levels. Further implementation of the use of distinct spectral sensitivity, for instance using light LOV domains [157] or drug-induced dimerisation, such as the knock sideways techniques [158], will be powerful. However, the initial focus must be on fully validating these systems to ensure that they function as the endogenous protein. This validation is facilitated at least for some of the components where highly specific antibodies are readily available for immunofluorescence imaging [159,160]. Comparative analysis that the cellular stability of the genome-tagged proteins, the steady-state expression levels and localisation dynamics closely follow that of the endogenous untagged version is critical. The realisation that widely used GFP derivatives (27 kDa) are relatively large proteins compared with YAP (70 kDa) and TAZ (55 kDa) is imperative. Some of these fluorescent proteins have additional dimerisation properties, which might combine with potential additional properties such as their ability to form biological condensates [161–163] as well as the ability to pass through the size filtering nuclear pore complexes [164]. Consequently, tagging Hippo pathway components might functionally impair YAP/TAZ and thus highlights that detailed design and characterisation must be carried out. The use of intra- or nano-bodies activatable optogenetic tools combining the specificity and orthogonality of intrabodies with the spatio-temporal precision of optogenetics might overcome some of these limitations [165–168]. Taken advantage of the full spectrum of these exciting techniques will be especially powerful to delineate the dynamics of the pathway, while also allowing to distinguish the intrinsic challenge within the Hippo pathway to determine between transcription dependent and independent mechanisms. We forsee that by using advanced experimental approaches, additional multilevel cellular regulation and integration centred on the Hippo pathway will become apparent in the years to come [55,56,58,59,145]. Realisations of these molecular couplings will provide fundamental insights into cellular processes while also providing new foundational therapeutic opportunities to target the challenging Hippo pathway.

## Perspectives

- Multilevel dynamic feedback is critical to safe-proofing fundamental cellular processes — the Hippo pathway is at the centre of this cellular decision-making process.
- It remains challenging to distinguish Hippo pathway transcription independent from dependent functions, as YAP/TAZ regulates more than 1000 genes [69,126,169,170].
- The use of newly developed technologies allows for establishing ‘moonlighting’ functions. For instance, what other substrates do STRIPAK and the range of Hippo pathway kinases have besides regulation of the core Hippo pathway? Spatio-temporal cooperation between components within the Hippo pathway and additional cellular components adds further complexity to the Hippo pathway. It is likely that in the years to come, additional layers of these feedback loops and synergistic multilevel regulation will be revealed and these discoveries are likely to be of therapeutic importance. It might therefore be useful to think about the pathway as a network instead of a linear pathway.

## Open Access Statement

Open access for this article was enabled by the participation of University of Edinburgh in an all-inclusive *Read & Publish* pilot with Portland Press and the Biochemical Society under a transformative agreement with JISC.

## Abbreviation

CDK: cyclin-dependent kinase
CTCF: CCCTC-binding factor
ECM: extracellular matrix
FAs: focal adhesions
GAPs: GTPase-activating proteins
LPA: lysophosphatidic acid
MMB: multiprotein Myb-MuvB
